# A Novel Variant in the *ACVRL1* Gene in a Patient with Cirrhosis and Hereditary Hemorrhagic Telangiectasia

**DOI:** 10.4274/tjh.galenos.2021.2020.0749

**Published:** 2021-08-25

**Authors:** Mehmet Baysal, Nihan Alkış, Hakan Gürkan, Ahmet Muzaffer Demir

**Affiliations:** 1 Bursa City Hospital, Clinic of Hematology, Bursa, Turkey; 2 Trakya University Faculty of Medicine, Department of Medical Genetics, Edirne, Turkey; 3 Trakya University Faculty of Medicine, Department of Hematology, Edirne, Turkey

**Keywords:** Hereditary hemorrhagic telangiectasia, ACVRL1 mutation, Cirrhosis, Epistaxis, Anemia

## To the Editor,

Hereditary hemorrhagic telangiectasia (HHT) is a rare bleeding disorder characterized by arteriovenous malformations (AVMs), telangiectasia, and bleeding episodes [1]. Pulmonary, hepatic, and cerebral AVMs may be seen in the course of the disease [2]. Mutations in the *ENG*, *ACVRL1*, and *SMAD4* genes were associated with HHT [3]. A 65-year-old man was admitted to our hospital with anemia and intermittent nose bleeding. Upon physical examination, telangiectasias were noticed on his face and nose. Further investigations in his work-up revealed hypochromic microcytic anemia with a hemoglobin level of 8 g/dL. Detailed laboratory analysis revealed iron deficiency anemia. In the upper gastrointestinal endoscopy performed for iron deficiency anemia, grade 1 esophageal varices were detected and intravenous iron carboxymaltose treatment was planned. His epistaxis severity score was 3.22, which can be categorized as mild bleeding [4].

**Figure 1:**
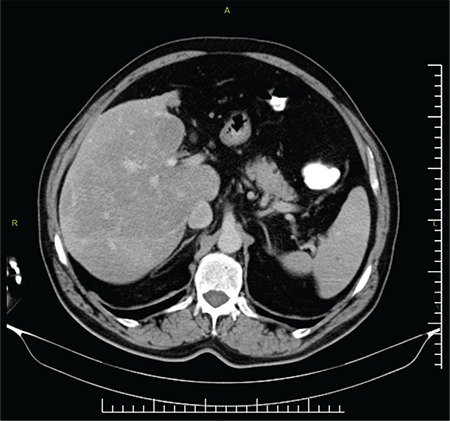
Computed tomography of the abdomen showed nodularity of the surface of the liver, a heterogeneous appearance of the liver parenchyma, and atrophy of the left liver lobe.

Family history revealed positive findings for nose bleeds and telangiectasia in his first-degree relatives and molecular genetic analysis was performed on a next-generation sequence analysis platform (NextSeq550-Illumina) using the QIAseq Targeted DNA Panel Kit (CDHS-14647Z-252-QIAGEN), which includes the *ACVRL1, ADAM17, ENG, GDF2, PTPN14, RASA1*, and *SMAD4* genes. Variant analysis was performed using QIAGEN Clinical Insight software. As a result of the bioinformatics analysis performed considering the ACMG-2015 criteria, the NM_000020.3(*ACVRL1*):c.1415G>A (p.Trp472Ter) variant was evaluated as pathogenic according to the PVS1, PM2, and PP3 rules (in silico analysis results - DANN score: 0.9944, GERP score: 4.4, MutationTaster: Disease causing). The *ACVRL1*:c.1415G>A variant was reported in the dbSNP database with reference number rs1555154144, but its clinical significance was not reported in the ClinVar or HGMD Professional 2020.3 databases. The minor allele frequency was not reported in the dbSNP, ExAC, or GnomAD_exome databases [5,6]. Computed tomography of the abdomen showed nodularity of the surface of the liver, a heterogeneous appearance of the liver parenchyma, and atrophy of the left liver lobe (Figure 1). No arteriovenous malformations were found in the liver and evaluation of the portal venous system was normal. Hepatitis virus markers, immunoglobulin levels, and autoimmune markers were normal. As the patient’s anamnesis was detailed, a history of regular alcohol consumption was noted and the patient was diagnosed with Child A liver parenchymal disease. A colonoscopic evaluation of the patient was also performed, and multiple small telangiectases were seen in the rectal mucosa. Local preventive measures and tranexamic acid were given for epistaxis and low-dose propranolol was started for grade 1 esophageal varices.

Gastric and hepatic manifestations of HHT are broad, and on rare occasions HHT can be associated with liver cirrhosis [7,8]. However, as in our case, HHT and alcohol intake have both caused and triggered liver cirrhosis. Our patient has stopped consuming alcohol and is being followed as an outpatient for both HHT and cirrhosis. Mutations in the *ACVRL1* gene occur more frequently in HHT type 2 patients, and according to the University of Utah mutation database there are 571 confirmed variants in the *ACVRL1* gene associated with HHT; our novel variation was not reported before [9]. Regardless of the age of the patient, HHT should be on the physician’s mind when evaluating a patient with telangiectasias and unexplained iron deficiency.
